# A metal ion orients SARS-CoV-2 mRNA to ensure accurate 2′-*O* methylation of its first nucleotide

**DOI:** 10.1038/s41467-021-23594-y

**Published:** 2021-06-02

**Authors:** Thiruselvam Viswanathan, Anurag Misra, Siu-Hong Chan, Shan Qi, Nan Dai, Shailee Arya, Luis Martinez-Sobrido, Yogesh K. Gupta

**Affiliations:** 1 Greehey Children’s Cancer Research Institute, University of Texas Health at San Antonio, San Antonio, TX USA; 2 Department of Biochemistry and Structural Biology, University of Texas Health at San Antonio, San Antonio, TX USA; 3 New England Biolabs, Ipswich, MA USA; 4 Texas Biomedical Research Institute, San Antonio, TX USA

**Keywords:** Multienzyme complexes, X-ray crystallography

## Abstract

The SARS-CoV-2 nsp16/nsp10 enzyme complex modifies the 2′-OH of the first transcribed nucleotide of the viral mRNA by covalently attaching a methyl group to it. The 2′-*O* methylation of the first nucleotide converts the status of mRNA cap from Cap-0 to Cap-1, and thus, helps the virus evade immune surveillance in host cells. Here, we report two structures of nsp16/nsp10 representing pre- and post-release states of the RNA product (Cap-1). We observe overall widening of the enzyme upon product formation, and an inward twisting motion in the substrate binding region upon product release. These conformational changes reset the enzyme for the next round of catalysis. The structures also identify a unique binding mode and the importance of a divalent metal ion for 2′-*O* methylation. We also describe underlying structural basis for the perturbed enzymatic activity of a clinical variant of SARS-CoV-2, and a previous SARS-CoV outbreak strain.

## Abstract (web summary)

The SARS-CoV-2 nsp16/nsp10 enzyme complex methylates the 2′-OH of the first nucleotide of the viral mRNA, converting the Cap-0 to Cap-1, which helps the virus to evade immune surveillance in the host cell. Here, the authors present the crystal structure of SARS-CoV-2 nsp16/nsp10 with the bound Cap-1 RNA nucleotide product and a post-release SAH containing structure.

## Introduction

RNA viruses employ a diverse set of protein assemblies to enzymatically modify the 5′-end of their genomes. This process, termed RNA capping, is essential for efficient production of viral proteins, protection of viral (v)RNA from host degradation, and evasion of the host innate immune responses—all of which enable viruses to thrive inside the host body^1^. In coronaviruses (CoVs), nonstructural protein 16 (nsp16) and its non-catalytic stimulator nsp10 assemble on the 5′-end of nascent mRNA to perform the last step of RNA cap modification—*S*-adenosyl-l-methionine (SAM)-dependent methylation of the 2′-OH on the first transcribed nucleotide base (N_1_), usually an adenine. This converts the status of RNA from Cap-0 (^me7^GpppA) to Cap-1 (^me7^GpppAm)^2,3^. Suppression of innate host antiviral response by Cap-1 through IFIT (interferon (IFN) signaling-induced proteins with tricopeptide repeat) proteins^4^ is thought to be a consequence of diminished binding of IFIT to Cap-1^5^. Genetic ablation of nsp16 enzymatic activity also leads to induction of type I IFN via the RNA sensor melanoma differentiation-associated protein 5^6^. Emerging evidence suggests that CoV disease 2019 (COVID-19) patients have elevated innate immune responses, causing hypercytokinemia^7^. Thus, structural elucidation of different stages of Cap-1 formation and modification will further our understanding of the 5′-RNA biology of CoVs and may inform the path to rational drug design.

In CoVs, nsp10 allosterically stimulates both 2′-*O-*ribose methylation of A_1_ base by nsp16 and *N*^7^ methylation of terminal guanine (G_0_) base of the unmethylated mRNA cap (G_0_pppA_1_) by nsp14^2,8,9^. These two different enzymatic activities would require rapid recycling of nsp10 from nsp16/nsp10 and nsp14/nsp10 complexes after 2′-*O* methylation of A_1_ and guanine-*N*^7^-methylation, respectively. In this work, we present two high-resolution crystal structures of the 2′-*O* methyltransferase enzyme complex (nsp16/nsp10 heterodimer) of severe acute respiratory syndrome CoV2 (SARS-CoV-2). These structures represent different stages of the catalytic cycle, i.e., pre- and post-release states of the mRNA cap product after 2′-*O* methylation. A structural comparison with the enzyme bound to the substrate mRNA cap reveals the nature of conformational changes that may occur during and after the catalysis. We show that a divalent metal ion binds to nsp16 and orients the mRNA in the catalytic pocket for accurate 2′-*O*-ribose methylation of the first transcribed nucleotide (A_1_) of the SARS-CoV-2 genome. We also show that a single mutation in a clinical variant of SARS-CoV-2 severely attenuates the enzymatic activity of nsp16. In contrast, the corresponding mutation in SARS-CoV enhances the activity of nsp16. Moreover, both mutants show opposite sensitivities to calcium ions. Our work thus suggests that the Cap-1-mediated immune avoidance could be modulated by mutations in nsp16 and/or different divalent metals in the host environment.

## Results and discussion

**Fig. 1:**
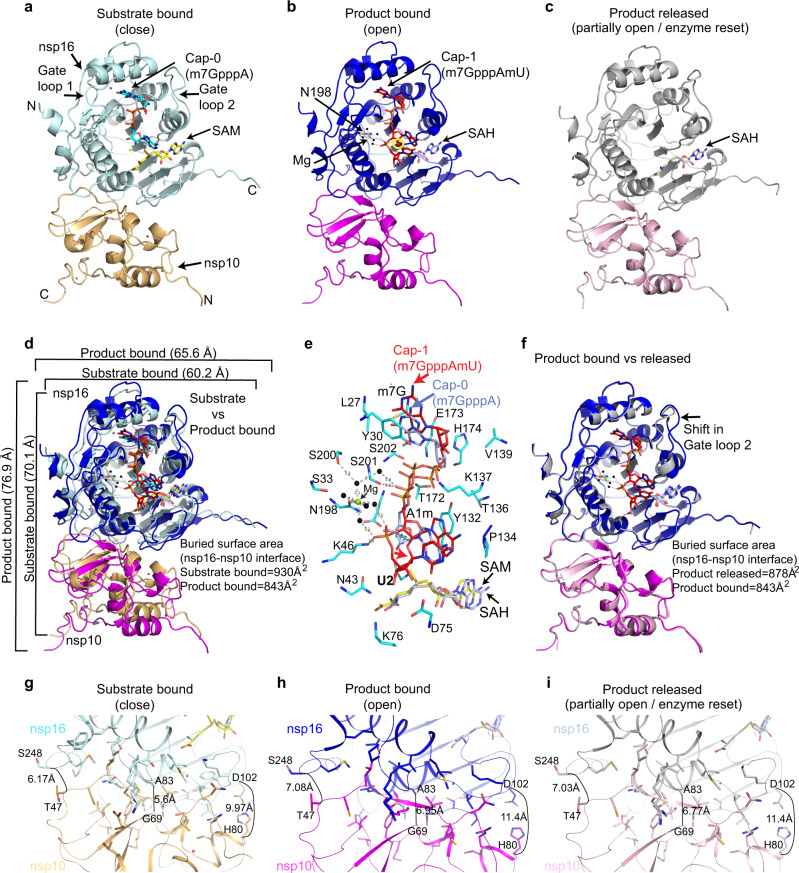
Structures of SARS-CoV-2 nsp16/nsp10 complexes. **a** The substrate (^me7^GpppA, cyan stick) and methyl donor *S*-adenosyl-l-methionine (SAM, yellow stick)-bound nsp16 (cyan)/nsp10 (orange) complex (PDB ID, 6WKS)^9^ represent a closed form. **b** The product (^me7^GpppAmU, red stick; byproduct *S*-adenosyl homocysteine [SAH, gray stick])-bound nsp16 (blue)/nsp10 (magenta) in an open state. A yellow circle shows the methylated ribose (2’-*O*-me) of N_1_ (A) base. **c** The SAH (gray)-bound nsp16 (gray)/nsp10 (pink) represents a partially open or enzyme reset state. **d** Secondary structure-based overlay of nsp16 in substrate- and product-bound states clearly shows the universal expansion of the enzyme upon 2’-O methylation. **e** A close-up view of Cap-1-binding and catalytic pocket of the product structure shows nsp16 residues (cyan sticks) interacting with Cap-1 (red). A positional change in orientation of the substrate (Cap-0, blue) from the “closed” structure determined previously^9^ is shown. **f** An overlay of the product (Cap-1)- and byproduct (SAH)-bound structures shows change in the orientation of gate loop 2. Reduction in buried surface area between nsp16/nsp10 in fully and partially open structures (compared to substrate-bound closed state) is shown (**g**–**i**).

We and others have previously resolved the structures of the SARS-CoV-2 nsp16/nsp10 enzyme complex in the presence of a Cap-0 analog (^me7^GpppA) and methyl donor SAM (Fig. 1a)^9,10^. Here we report two structures of the nsp16/nsp10 heterodimer complex in the presence of a cognate RNA product (Cap-1) that consists of N_1_ and an adjoining N_2_ base (^me7^GpppAmU), and a byproduct of the methylation, *S*-adenosyl-l-homocysteine (SAH), resolved to 2.3 and 2.5 Å, respectively (Fig. 1b, c and Supplementary Table 1). These structures were solved by a molecular replacement method using the previously determined Cap-0 (^me7^GpppA)/SAM structure (PDB ID: 6WKS, hereafter referred to as the “substrate structure”) as a search model^9^. Cap-1 RNA and SAH were unambiguously identified in the difference omit maps (Supplementary Fig. 1a–c). nsp16 adopts a canonical methyltransferase fold with a central β-sheet flanked by two α-helices on one side and three on the other, similar to the substrate Cap (Cap-0)-bound structure^9^ but with a notable difference as described below.

### nsp16/nsp10 undergoes breathing motion during 2′-*O* methyl transfer

A structural comparison of nsp16/nsp10 in substrate (^m7^GpppA and SAM)-, product (^m7^GpppAmU and SAH)-, and byproduct (SAH only)-bound structures revealed notable conformational changes in three complexes (Fig. 1). Most strikingly, we observed an overall expansion of the nsp16/nsp10 complex in the product structure as compared to the substrate-bound enzyme (~6.8 Å in one dimension and ~5.4 Å in the other), although the central β-sheet remains largely unperturbed (Fig. 1d, e and Supplementary Fig. 1i). A positional shift in the Cap-1 analog also occurs, so that the 2′-*O*-me group on the A_1_ base now pushes the SAH outward with the sulfur atom pointing away from the 2′-*O*-me moiety and the carboxy tail of SAH rotates 180° around C_β_ of the SAH (Fig. 1e and Supplementary Fig. 1a–h, j). A comparison of the product and byproduct structures reveals no major changes in protein conformations, except for an inward shift in gate loop 2 (Fig. 1f). The position of SAH in the product and byproduct structures remains essentially the same, except for the carboxy tail, which rotates back in the byproduct structure to assume the original orientation as in the substrate structure (Fig. 1b–d and Supplementary Fig. 1b, g, h). These changes suggest that the conformation of the SAH-bound enzyme represents a resetting for the next round of catalysis.

The surface area buried (BSA) at the nsp16/nsp10 interface is significantly smaller (843 Å^2^) in the product complex than the substrate structure (930 Å^2^) (Fig. 1d, g). This reduction is less pronounced (BSA = 878 Å^2^) in the byproduct structure (Fig. 1f). As a result, the heterodimeric interface in the product structure (and to some extent the byproduct structure) is widened by ~1.5 Å at one end, ~0.9 Å at the other, and ~1.35 Å in the center (Fig. 1g–i). The much-relaxed heterodimeric interface in the product structure appears to be a result of overall widening of the enzyme, which is triggered by a single 2′-*O* methylation event. The enzyme appears to go into a “breathing motion” during catalysis, wherein the substrate-, product-, and byproduct-bound states represent fully closed, open, and partially open (product released/enzyme reset) states, respectively (Fig. 1). The product RNA Cap, Cap-1 with adjoining uracil as N_2_ base at the 3′-end, is accommodated within a deep and elongated pocket constituted by the nsp16 residues on one side of the central β-sheet and the two gate loops (gate loop 1 [amino acids 20–40] and gate loop 2 [amino acids 133–143]). The byproduct SAH in both structures binds similarly in a cavity at the C-terminal side of the parallel β-strands, except for different orientations of their carboxy tails (Supplementary Fig. 1a–c, g–h).

A closer examination of the RNA cap-binding regions of nsp16 in the product (Cap-1) and substrate (Cap-0) structures provides some insights on the mechanics of RNA product release after catalysis (i.e., 2′-*O* ribosyl methylation). The RNA cap-binding groove of nsp16 in Cap-0 structure spans an overall distance of ~16.4 Å from L27 to K46^9^. The L27 forms a hydrogen bond through its amide nitrogen with the O6 of terminal G_0_ base, whereas K46 forms a hydrogen bond with the 3′-OH of the target A_1_ base. The RNA cap in this groove is stabilized by an extensive network of electrostatic interactions with nsp16 residues that are arranged in three layers of semi-circles around the entire cap region. In the bottom layer, the side chains of K46, D130, Y132, P134, K170, and E203 stabilizes the target A_1_ base. The middle layer of interaction network that stabilizes the inverted triphosphate moiety of RNA cap consists of S201, S202, T172, K137, and T136 of nsp16. The nsp16 residues at the top semi-circle (L27, Y30, E173, and H174) stabilizes the terminal G_0_ base through electrostatic and stacking interactions^9^. In the Cap-1 structure, the RNA cap-binding groove extends up to ~18.11 Å and the distance between different regions of two loops (Gate loop 1 and 2) that surround the RNA cap also increases up to ~2.8 Å from their respective positions in Cap-0 structure, concomitant to overall widening of the enzyme upon Cap-1 formation (Supplementary Table 2). Consequently, almost all nsp16 residues (described above) that interact with product Cap-1 move up to ~1.61 Å away from their respective positions in the Cap-0 structure, thus creating a weaker Cap-1-nsp16 interface (Fig. 1e, Supplementary Fig. 1e, and Supplementary Table 2). Consistently, weaker affinity of nsp16/nsp10 to Cap-1 (*K*_D_ = 121 µM) than Cap-0 (*K*_D_ = 97 µM), as observed in our previous work^9^, further corroborate our structural findings presented here.

The most significant deviations in the positioning of nsp16 residues around Cap-1 are observed in K46 and E203, part of the catalysis tetrad “K_46_-D_130_-K_170_-E_203_ (or KDKE motif)” and N198, the residue that directly ligates to a metal ion (see details in next section). The K46 side chain, which is at 3.39 Å distance from the target 2′-OH in the Cap-0 structure, moves away and resides at 5.04 Å from the 2′-*O*-me in the product structure (Fig. 1e and Supplementary Fig. 1e). In the Cap-0 structure, the side chain of N198 forms electrostatic interaction through the 3′-OH of the target A_1_ base, whereas it directly interacts with Mg^2+^, which in turn coordinates to phosphoryl oxygens of the U_2_ base in the product structure (Fig. 1e and Supplementary Fig. 1e, j). Thus, the new protein–metal–RNA interactions occuring through N198 in Cap-1 could not be formed due to the lack of downstream RNA (U_2_) in nsp16/nsp10 structure with Cap-0 analog (Supplementary Fig. 1j and Supplementary Table 2).

A previous biochemical study on the Middle East respiratory syndrome CoV nsp16/nsp10 complex observed the highest stability of nsp16/nsp10 heterodimer in the presence of 100 µM SAM, a concentration close to intracellular SAM levels, but dissociation of the heterodimer at the lower intracellular concentration (20 µM) of SAH, the byproduct of methylation reaction^11^. As nsp10 is required for allosteric stimulation of the enzymatic activities of nsp14 (for *N*^*7*^-methylation of G_0_) and nsp16 (for 2′-*O*-ribose methylation of A_1_) of the mRNA cap, nsp10 must rapidly dissociate from nsp16/nsp10 and nsp14/nsp10 complexes after each round of catalysis for efficient turnover. The overall expansion in nsp16/nsp10/product Cap-1 complex and the accompanied reduction in buried surface area between the two subunits may be the structural basis of nsp16 and nsp10 dissociation post 2′-*O*-ribose methylation (Fig. 1d, h). Thus, the Cap-1 structure may represent a post-methylation state preceding the release of SAH and product RNA, and the dissociation of nsp16/nsp10 heterodimer. Future studies should determine the exact order of disassembly of the complex.

### Metal dependency for 2′-*O* methylation by SARS-CoV-2 and its variants

**Fig. 2:**
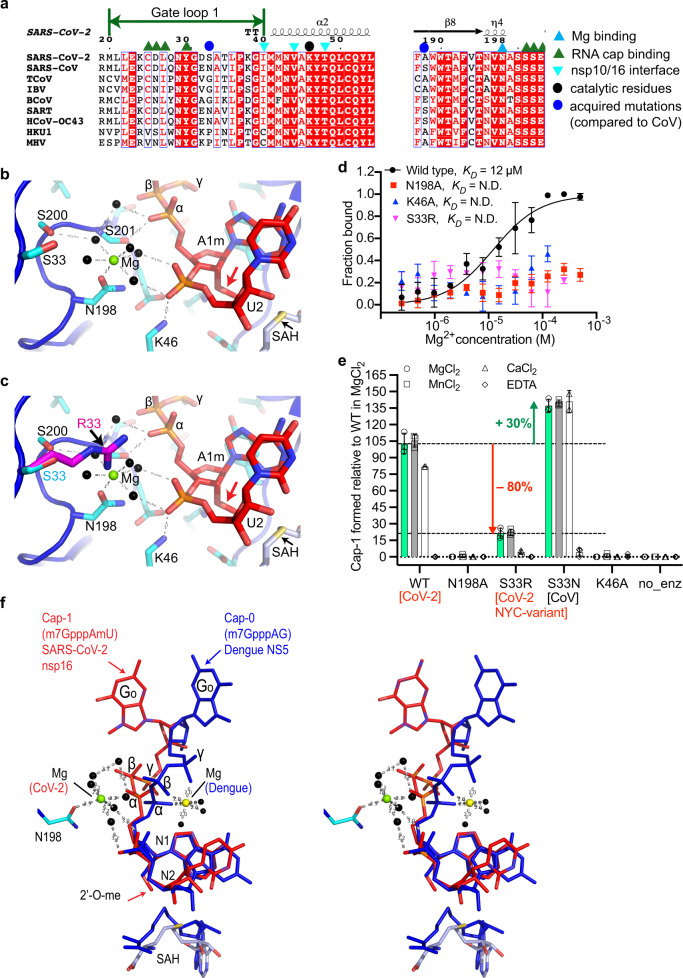
Metal dependency of nsp16/nsp10 and its clinical variant for 2’-*O* methylation. **a** Alignment of nsp16 from different CoV representing the α, β, γ sub-classes. Blue sphere denotes S33R, which locates in gate loop 1; S33 is an asparagine (N) in SARS-CoV; S33R is a clinical nsp16 variant of SARS-CoV-2; cyan triangle, N198 that coordinates Mg^2+^; and black sphere, catalytic lysine (K46). **b** A Mg^2+^ ion (green sphere) coordinates with five water molecules (black spheres) at the nucleic acid face and side chain of N198 on the opposite face. Two water molecules hold the phosphoryl oxygens of U_2_. Red arrow; 2’-*O*-methylated ribosyl of A_1_ base. **c** Side chain of arginine (magenta, modeled) at the S33 (cyan) position intrudes into the Mg^2+^ pocket and may displace Mg^2+^ or disrupt the Mg^2+^/water network. **d** Binding of Mg^2+^ to nsp16/nsp10 as derived from three independent MST experiments (*n* = 3) with 1 SD shown as error bars (N.D., not determined). **e** Quantitative measurement of Cap-1 formation by nsp16/nsp10 enzymes (±metals, ethylenediaminetetraacetic acid [EDTA]) derived from LC/MS. Error bars indicate range of data points from three independent experiments (*n* = 3, except *n* = 2 for WT and S33N in CaCl_2_) normalized to WT in MgCl_2_. Center of error bars is 102.8%, 105.4%, and 81.9% for WT in MgCl_2_, MnCl_2_, CaCl_2_; 21.4%, 22.4%, and 4.3% for S33R; and 137.1%, 139.6%, and 140.8% for S33N, respectively. N198A, K46A, and EDTA reactions show negligible Cap-1 formation. Spheres and green bars, MgCl_2_; squares and gray bars, MnCl_2_; triangles and white bars, CaCl_2_; and diamonds, EDTA. Source data are provided as a Source Data File. no_enz, reaction devoid of nsp16/nsp10 enzyme. **f** A stereo view of an overlay of the N_1_ and N_2_ bases, and SAH of SARS-CoV-2 nsp16/nsp10 (red) and dengue NS5 (blue; PDB ID: 5DTO). RNA caps show entirely different orientations of the terminal base of the cap (^me7^G), two phosphates (β and γ) and Mg^2+^ ions. Mg^2+^ (in dengue, yellow sphere) stabilizes the three phosphates, whereas in SARS-CoV-2 (green sphere) it indirectly (water-mediated) stabilizes the phosphate of N_2_ on one side and engages N198 of nsp16 on the opposite side.

We also observed unambiguous electron density in omit maps at the interface of gate loop 1, a loop between the β8 and β9 strands, and the phosphate moiety of uracil (U_2_), the N_2_ base downstream to the RNA Cap (Fig. 1e and Supplementary Fig. 1a, c). The features of this density suggested a divalent metal ion coordinating with water molecules. Metal ions stabilize nucleic acid substrates in addition to acting as catalytic agents in enzymatic reactions. In other positive single-stranded RNA viruses (e.g., 2′-*O* MTase such as dengue NS5^12^), a magnesium ion stabilizes the RNA cap by coordinating with the inverted triphosphate moiety from the solvent-exposed side of the RNA cap (Fig. 2f). A magnesium ion in nsp16 of the previous CoV outbreak strain (PDB ID: 2XYR) binds to a remote site constituted by T58 and S188 located at the opposite face of the catalytic pocket (Supplementary Fig. 2)^13^. A direct binding of metals in the substrate/catalytic pocket and their role in 2′-*O* MTase activity of the CoVs nsp16, including SARS-CoV-2, has not been previously suspected.

As our protein purification buffers and crystallization solutions contain magnesium and calcium salts, respectively, we fit these metals into the additional electron density, but could refine the product structure with better statistics when Mg^2+^ was modeled along with its coordinating water molecules (B factor = 52.5 Å^2^ and 73.3 Å^2^ for Mg^2+^ and Ca^2+^, respectively) in this density. As modeled, Mg^2+^ is further stabilized by direct interaction with the side chain of an invariant N198 and assumes a near-ideal octahedral geometry with coordinating water molecules (Fig. 2a, b and Supplementary Fig. 1d, e, j). Consistently, the wild-type (WT), but not the N198A mutant of nsp16/nsp10 binds Mg^2+^ with high affinity (Fig. 2d). A direct interaction of protein to Mg^2+^ and its orientation in the Cap-binding pocket is unique to SARS-CoV-2 (Fig. 2c). For example, in dengue NS5, Mg^2+^ is exposed to solvent and cross-links the phosphate groups of the RNA cap without directly ligating to the protein^12^, whereas it directly binds to nsp16 (through N198) and the phosphate of the U_2_ base (water-mediated) without ligating to the inverted triphosphate moiety of the mRNA cap in SARS-CoV-2 (Fig. 2f). Although the overall fold of the MTase domains of dengue NS5^12^ and SARS-CoV-2 nsp16^9^ is similar, the loops connecting the secondary structural elements of NS5 MTase are longer and have a slightly different orientation than nsp16. The sequence identity between the two MTases is low (~13%) and there is no obvious similarity to the gate loop 1 and N198 of nsp16 to the corresponding region of nsp16. Thus, the role of magnesium ion for 2′-*O* methylation of viral mRNAs appears to be architectural, as it helps to maintain the RNA cap in a certain confirmation amenable to proper catalysis of the target base.

Our previous work revealed the basis of target specificity of nsp16 for adenine nucleotide at the N_1_ position^9^. We and others postulated that K170 (the second lysine of the c catalytic tetrad) acts as a general base to facilitate methyl transfer from SAM to 2′-OH of the A_1_ nucleotide in SARS-CoV-2^9^ and other CoVs^3,13^. The product structure allowed us to examine the roles of K170 and K46 (first lysine of the KDKE catalytic tetrad) in detail. The side chains of K170 and K46 form hydrogen bonds with the 2′-*O* of the product (i.e., methylated ribosyl of A_1_ and phosphoryl oxygen of U_2_ base, respectively, Fig. 2b and Supplementary Fig. 1d–f). The phosphoryl oxygens of U_2_ also interact with water molecules that coordinate with Mg^2+^. Mutation of K46 and N198 to alanine completely abolished Mg^2+^ binding and catalytic activity of nsp16/nsp10 (Fig. 2d, e). This is consistent with the network of side chains of K46, K170, and N198, which we identify here as important for catalysis. Such an arrangement correctly positions the RNA cap in the catalytic pocket to both ensure efficient 2′-*O* methylation of A_1_ base and prevent unintended methylation of the adjoining U_2_ base by restricting its movement or misalignment during catalysis of the A_1_ base. The U_2_ nucleotide, although largely exposed to solvent and showed some deviation in the geometry, resides at ~4 Å distance from the side chain of an invariant D75 residue of nsp16, which is located at the tip of the loop that connects the β2 strand and α3 helix (Fig. 1e and Supplementary Fig. 1d, e).

The frequency of mutations in SARS-CoV-2 is much lower (about half) than other RNA viruses, yet 12,000 mutations have been identified in the SARS-CoV-2 genomes^14^. Although higher disease transmissibility and infectivity by variants of concerns is associated with high-frequency mutations in spike protein (e.g., D614G)^15,16^, such analyses for mutations in nonstructural proteins are yet to be emerged. Previously, we mapped the acquired mutations that have been identified in SARS-CoV-2 nsp16 (compared to SARS-CoV nsp16) on its structure and postulated their potential roles in RNA binding and/or catalysis^9^. Among these, residue S33 in gate loop 1 is particularly noteworthy, given the high occurrence of the S33R (20755:A > C) mutation in SARS-CoV-2 strain associated in the New York City outbreak^17^, some Latvian SARS-CoV-2 isolates^18^, and its mutation to an asparagine (S33N) in a previous CoV outbreak strain (Fig. 2a). The side chain of S33 resides 6.3 Å and 9.6 Å away from the Mg^2+^ and phosphoryl oxygens of the U_2_ base in the product structure, respectively (Fig. 2b). The side chain of the arginine in S33R, as we modeled in Fig. 2c, may intrude into this pocket to reduce these distances by ~4.18 Å, thus disrupting the coordination of Mg^2+^, which would in turn disorient the target base (A_1_) in the catalytic pocket. A shorter side chain of asparagine would be less intrusive and may provide additional contacts to a divalent metal ion, strengthen RNA binding, and therefore enhance 2′-*O* methylation.

To verify these models, we employed liquid chromatography-mass spectrometry (LC/MS) to measure Cap-1 formation by the SARS-CoV-2 nsp16/nsp10 and variant enzymes, and their dependence on various metal ions. Consistent to magnesium-binding affinity, where mutants N198A, K46A, and S33R exhibited negligible Mg^2+^ binding (Fig. 2d), mutating N198 (which directly interacts with magnesium) and K46 (which stabilizes the phosphate of U_2_) to alanine completely abolished the enzymatic activity of nsp16/nsp10 (Fig. 2e). Strikingly, the activity of S33R mutant decreased by ~80%, further validating our structural interpretation about this clinical variant (Fig. 2c–e). In contrast, the S33N mutation resulted in 30% increased activity, suggesting that the SARS-CoV nsp16, which has N at this position may have superior 2′-*O* methylation capability compared to SARS-CoV-2 nsp16 (Fig. 2e). Moreover, the SARS-CoV-2 nsp16 shows indistinguishable 2′-*O* methyltransferase activity in the presence of magnesium and manganese, but a 20% loss in the presence of calcium. The S33N mutant showed no preference for any of the three divalent ions tested (Mg^2+^, Ca^2+^, and Mn^2+^). However, S33R showed comparable activity in the presence of Mg^2+^ and Mn^2+^ (although 80% less than the WT), but residual enzymatic activity in the presence of Ca^2+^ (Fig. 2e).

The unique role of a divalent metal ion in SARS-CoV-2 nsp16 appears to be architectural (Fig. 2f), yet it is essential for accurate and efficient 2′-*O* methylation of the first transcribed base of the SARS-CoV-2 genome. With Cap-1’s important role in evading host innate immune response, the dependence and preference of nsp16/nsp10 for divalent metal ions also suggests that an imbalance in cellular metal concentrations could influence host innate immune response to infections by various CoVs. In support of this hypothesis, hypocalcemia is considered as a strong predictor of in-hospital COVID-19 deaths^19,20^, and severely ill COVID-19 patients had significantly lower magnesium levels in the blood^21^. One possibility is that the sub-optimal cap 2′-*O* methylation activity of the nsp16/nsp10 variants, together with altered levels of divalent metals, could trigger excessive immune response and cause hypercytokinemia in a subpopulation of COVID-19-positive patients. Future studies should determine the direct correlations between RNA capping, metal levels in the host cellular milieu, and innate immune response.

## Methods

### Protein expression and purification

The nsp16 (NCBI reference sequence YP_009725311.1) and nsp10 (NCBI reference sequence: YP_0009725306.1) of the seafood market pneumonia SARS-CoV-2 isolate Wuhan-Hu-1 (NC_045512) were cloned into a duet vector. The *Escherichia coli* strain BL21 (DE3) capable of co-expressing nsp16 and nsp10 protein complex was induced with 0.4 mM isopropyl β-d-thiogalactopyranoside at OD_600_ = 0.6 followed by continued incubation of the cultures for 14 h at 18 °C. Cells from 1 L culture were collected by centrifugation at 8983 × *g* for 20 min and re-suspended in ice-cold lysis buffer (25 mM Tris-HCl pH 8.0, 0.5 M NaCl, 0.1 mM Tris(2-carboxyethyl)phosphine hydrochloride (TCEP), 10% glycerol) supplemented with a protease inhibitor tablet (Pierce). Cell lysis was accomplished using a microfluidizer (Analytik, UK) and the soluble fraction was separated by centrifugation at 158,000 × *g* for 40 min. The clarified soluble fraction, after passing through a 0.22 µm filter, was loaded on to a Nuvia IMAC column (Bio-Rad) pre-equilibrated in binding buffer containing 25 mM Tris-HCl pH 8.0, 0.2 M NaCl, 0.1 mM TCEP, 5% glycerol. The proteins were eluted by increasing the concentration of imidazole from 0 to 1.0 M. After proteolytically removing the poly-histidine tag and passing the sample through a second IMAC column, the nsp16/nsp10 protein complex was purified by successive passage through HiTrap heparin (heparin buffer A, 25 mM Tris-HCl pH 8.0, 0.05 M NaCl, 0.1 mM TCEP, 5% glycerol; heparin buffer B, 25 mM Tris-HCl pH 8.0, 2.0 M NaCl, 0.1 mM TCEP, 5% Glycerol), and Superdex 75 (buffer, 25 mM Tris-HCl pH 8.0, 0.5 M NaCl, 0.1 mM TCEP, 10% glycerol and 5 mM MgSO_4_) columns. The purified nsp16/nsp10 complex eluted from the Superdex 75 column was concentrated to 5 mg/mL and used immediately for subsequent biochemical and/or crystallographic studies. We used the same method for all mutant nsp16/nsp10 enzymes reported in this study. We introduced each single point mutation (S33R, S33N, K46A, and N198A) in the nsp16/nsp10 plasmid by site-directed mutagenesis using the primers listed in Supplementary Table 3.

### Crystallization, X-ray data collection, and structure determination

The initial nsp16/nsp10 complex was grown by the sitting drop vapor diffusion in a crystallization solution 10% (v/v) of 2-propanol, 0.1 M MES/NaOH pH 6.0, 0.2 M calcium acetate. After three to four rounds of optimization by varying pH, precipitant, and salt concentrations, we grew larger crystals amenable to synchrotron radiation. We soaked these crystals with an RNA analog representative of the Cap-1 structure (^me7^GpppA_(2′-O-me)_U) at 2 mM final concentration for 30 min at 20 °C. The crystals were cryo-protected by serial soaks in a solution containing the original mother liquor and increasing concentrations (0–20% v/v) of ethylene glycol and then flash-frozen in liquid nitrogen. Crystals of the nsp16/nsp10/Cap-1/SAH and nsp16/nsp10/SAH complexes diffracted X-rays to 2.3 and 2.5 Å resolution with synchrotron radiation, respectively (Supplementary Table 1). Both crystals belong to the space group P3_1_21 with similar unit cell dimensions *a* = *b* = 184 Å, *c* = 57 Å, *α* = β = 90°, and *γ* = 120°, and with one nsp16/nsp10 heterodimer per asymmetric unit. All data (measured at wavelength 1.07 Å) were indexed, integrated, and scaled using X-ray detector software, aimless, and various ccp4 suite programs integrated into the Rapid automated processing of data  pipeline at the NECAT-24ID beamline^22^. The structure was solved by molecular replacement using a Cap-0 ternary complex of nsp16/nsp10/SAM (PDB ID: 6WKS)^9^ structure as a template in Phaser^23^. The resulting maps indicated unambiguous electron densities for RNA Cap-1 and SAH. Ligand topologies and geometrical restraints were generated using PRODRG (http://prodrg1.dyndns.org), GRADE (http://grade.globalphasing.org), and eLBOW (Phenix)^23^ programs. We iteratively rebuilt and refined the model with good stereochemistry using the programs Coot^24^ and Phenix^22^ (Supplementary Table 1). All figures of structural models were generated using Pymol (The PyMOL Molecular Graphics System, Version 2.0 Schrödinger, LLC). The final figures were prepared using Adobe Illustrator (version 2021).

### Determining affinities of WT and mutant nsp16/nsp10 binding to magnesium

We used microscale thermophoresis (MST) to derive equilibrium dissociation constants (*K*_D_) for protein–metal interactions. A detailed protocol has been published^9^. Briefly, 20 µM of each enzyme complex was labeled by incubating with dye solution (60 µM) in the labeling buffer at room temperature for 30 min. The magnesium stock was prepared in the MST reaction buffer (20 mM HEPES pH 7.5, 150 mM NaCl, 0.5% glycerol, and 0.05% Tween 20) and twofold serial dilutions (from 2 mM stock) were made in 12 steps. The labeled protein (20 nM) was equally mixed into each ligand reaction (ligand concentration ranges 500 nM to 1 mM). The final reaction mixtures were loaded and measured on a Monolith NT.115 instrument (NanoTemper Technologies) at 25 °C. The results shown here are from three independent experiments. Data were fitted by a single-site binding model in GraphPad Prism (GraphPad Software, San Diego, CA).

### Enzyme activity assay

We used a LC/MS-based method (reported earlier)^9^ for quantitative measurement of Cap-1 formation by nsp16/nsp10 enzymes in the presence of various metals. Briefly, 0.1 µM of enzymes were allowed to react with 1 µM ^me7^GpppA-capped 25 nt RNA in a buffer (50 mM Tris-HCl pH 8.0, 5 mM KCl, 1 mM dithiothreitol, 0.2 mM SAM) supplemented with 1 mM MgCl_2_ or MnCl_2_, or CaCl_2_ or 5 mM EDTA. The reactions were incubated at 37 °C for 30 min, stopped by heating at 75 °C for 5 min in the presence of 5 mM EDTA, and were subjected to LC/MS intact mass analysis. Nucleic acids in the samples were separated using a Thermo DNAPac™ RP Column on a Vanquish Horizon UHPLC System, followed by mass determination using a Thermo Q-Exactive Plus mass spectrometer. The raw data were deconvoluted using Promass HR (Novatia, LLC). The deconvoluted mass peak ratios between reactants and the expected products were used to estimate the percentage of 2′-*O* methylation. Results shown in Fig. 2e are average of three independent experiments (*n* = 3) normalized to a WT dataset consisting of seven data points. Source data are provided as a Source Data File. The RNA substrate used in this assay is 5′-^me7^GpppAUAGAACUUCGUCGAGUACGCUCAA-[6-FAM]-3′.

### Reporting summary

Further information on research design is available in the Nature Research Reporting Summary linked to this article.

## Supplementary information

Supplementary Information

Reporting summary

## Data Availability

The information about coding sequences of nsp16 (NCBI reference sequence YP_009725311.1) and nsp10 (NCBI reference sequence: YP_0009725306.1) of the seafood market pneumonia SARS-CoV-2 isolate Wuhan-Hu-1 (NC_045512) used in this study is available at NCBI. Files for atomic coordinates and structure factors were deposited in the Protein Data Bank (PDB) under accession codes 7LW3 (product bound) and 7LW4 (SAH bound). All other relevant data are available from the corresponding author. Source data are provided with this paper.

## Source data

Source data
