# Supplementary material for: A metal ion orients SARS-CoV-2 mRNA to ensure accurate 2′-O methylation of its first nucleotide
Source: Nat Commun. 2021 Jun 2;12:3287. doi: 10.1038/s41467-021-23594-y (PMC8172916; doi:10.1038/s41467-021-23594-y)
Supplement: Supplementary file 1 — Supplementary Information [file 41467_2021_23594_MOESM1_ESM.pdf]

SUPPLEMENTARY INFORMATION

A metal ion orients SARS-CoV-2 mRNA to ensure accurate 2'-*O* methylation of its first nucleotide

Thiruselvam Viswanathan[1,2], Anurag Misra[1,2], Siu-Hong Chan[3], Shan Qi[1,2], Nan Dai[3], Shailee Arya[1], Luis Martinez-Sobrido[4], Yogesh K. Gupta[1,2,*]

[1] Greehey Children's Cancer Research Institute, University of Texas Health at San Antonio, 8403 Floyd Curl Drive, San Antonio, TX 78229, USA

[2] Department of Biochemistry and Structural Biology, University of Texas Health at San Antonio, 7703 Floyd Curl Drive, San Antonio, TX 78229, USA

[3] New England Biolabs, 240 County Road, Ipswich, MA, 01938, USA

[4] Texas Biomedical Research Institute, San Antonio, TX, 78227, USA

[*]Corresponding author:

Y.K.G email: guptay@uthscsa.edu

These authors contributed equally: Thiruselvam Viswanathan, Anurag Misra

This document contains the following:

Supplementary Figure 1-2
Supplementary Table 1-3
Supplementary References

Supplementary Figure 1

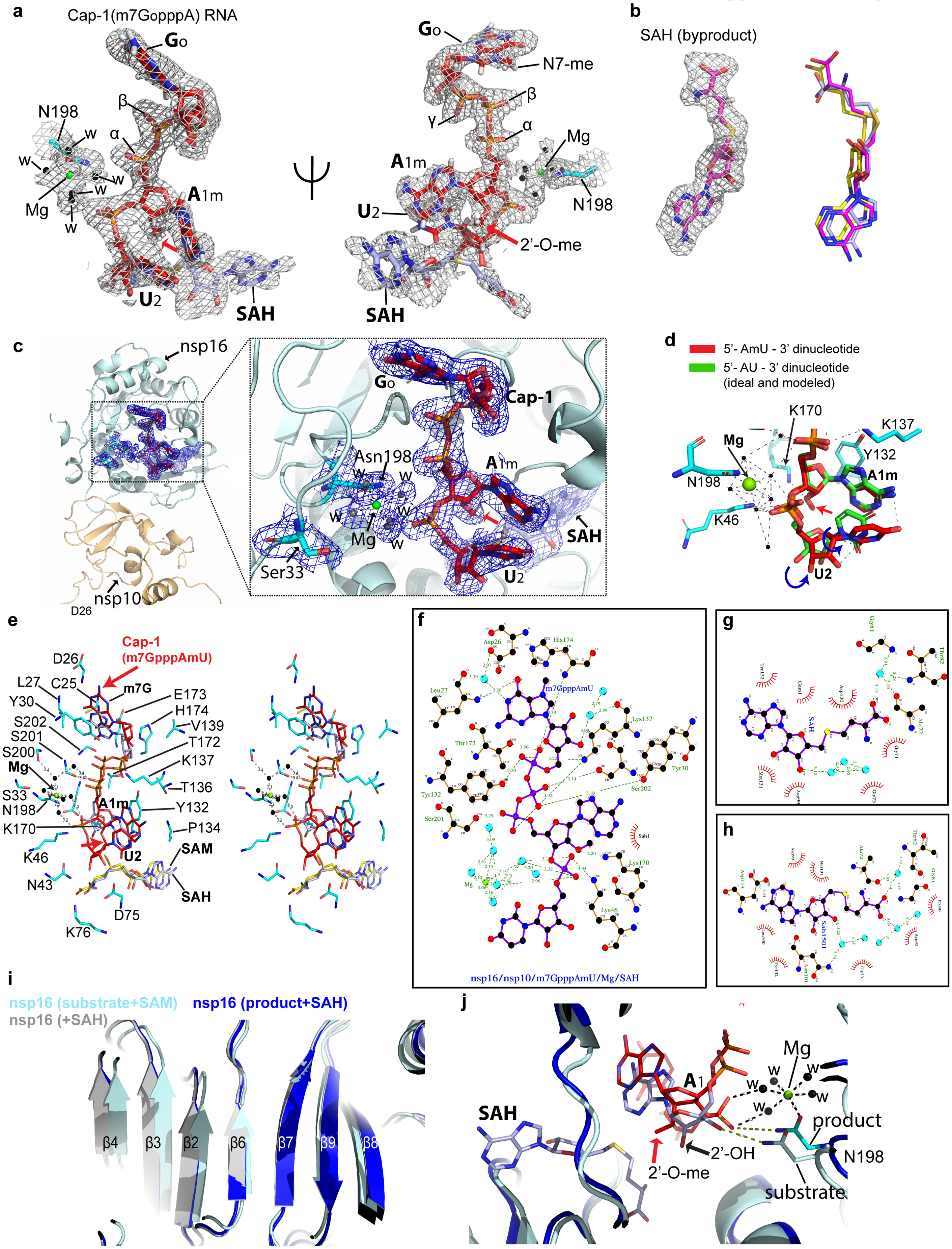

Supplementary Figure 1. a, A close-up view of the product (^{m7}GpppAmU, red stick), SAH (grey stick), magnesium (green sphere), water (W, black spheres), and N198 binding. The Fo-Fc electron density omit map for these ligands contoured at 2.7σ is shown as a grey mesh. To minimize the possibility of bias introduced by ligands, all ligands were excluded from refinement and phase calculations. Positions of the methyl group attached to the acceptor moiety (2'-O in ribose of the target nucleotide A_{1m}) is depicted by red arrow. **b,** The Fo-Fc electron density omit map for SAH (magenta stick) in byproduct (nsp16/nsp10/SAH) structure contoured at 2.7σ is shown as a grey mesh. Right panel, an overlay of SAM (yellow stick) in Cap-0, SAH (magenta stick) in byproduct, and SAH (grey stick) in Cap-1 structures. **c,** Final structure of nsp16 (cyan)/nsp10(orange)/$^{me7}G_o$pppAmU (red)/SAH (grey)/ Mg^{2+} (green)/Mg^{2+}-coordinating waters showing 2Fo-Fc map (blue mesh) contoured at 1.0σ. **d,** An overlay of an A in an ideal AU (green) dinucleotide over Am base in the product structure shows deviated geometry (outward motion, blue arrows) of the U2 base, suggestive of a state preceding the product's release. **e,** A stereo view of nsp16 residues (cyan) that directly interact with Cap-1 RNA (red) and Mg^{2+} (green sphere) and water (black sphere). **f,** Protein-ligand interaction network of nsp16/nsp10/Cap-1 (^{m7}GpppAmU) and SAH in product (**g**), and SAH-bound (**h**) nsp16/nsp10 structures. The green dashed lines represent hydrogen bonding, and the cyan spheres represent water molecules. These figures were generated using the LigPlot+ program[1]. **i,** A secondary structure-based superposition of nsp16 in all three structures show good overlay of the central β-sheet in all three structures. The regions flanking the central core in nsp16 and the entire nsp10 universally expands (relative to substrate/SAM-bound form, cyan) to assume a more relaxed (or fully open state) in the product-bound form (blue). The structures of product plus SAH and only SAH-bound enzymes do not deviate except in the gate loop region. **j,** An overlay of the substrate (light cyan) and product (blue) structure is shown with their respective Caps (Cap1U as red stick in product, grey stick; Cap-0 in substrate structures). In the absence of Mg^{2+} in the substrate structure, the side chain of N198 coordinates with 3'-OH of the N1 base whereas in the product structure it coordinates with the phosphoryl oxygen of the N2 base and Mg^{2+} ion.

Supplementary Figure 2

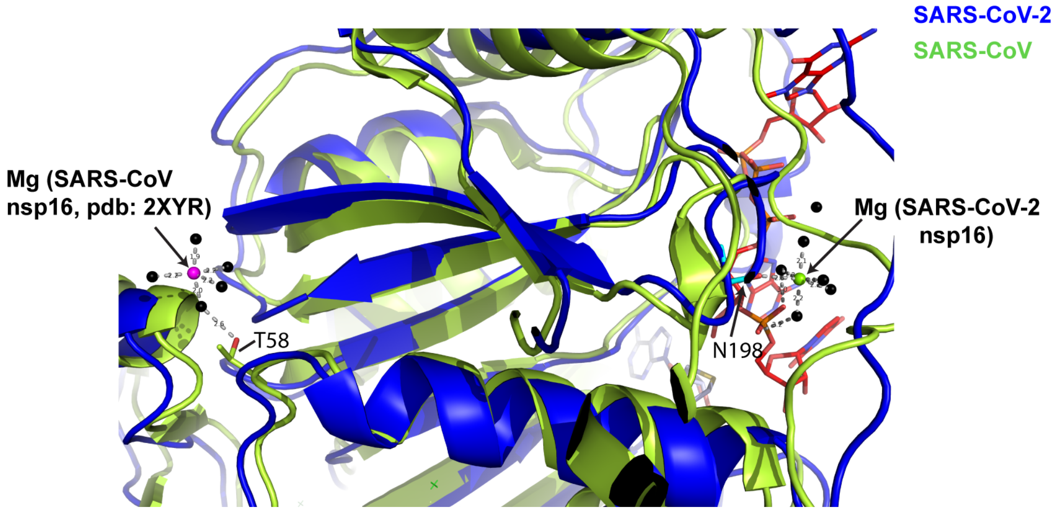

Supplementary Figure 2. An overlay of nsp16 of SARS-CoV-2 (blue ribbons) and SARS-CoV (green ribbons) shows binding of a magnesium ion to different regions of nsp16. In SARS-CoV-2, a magnesium (green sphere) directly interacts with N198 (cyan stick) whereas in SARS-CoV, it (magenta sphere) binds to the back side of nsp16 and interacts with T58 (green stick) through an oxygen from a water molecule.

Supplementary Table 1

Data collection and refinement statistics (molecular replacement)

		nsp16/nsp10/m7GpppAmU (PDB ID: 7LW3)	nsp16/nsp10/SAH (PDB ID: 7LW4)
**Data Collection**		NECAT-24ID, APS	NECAT-24ID, APS
Wavelength		1.071	1.071
Resolution range (Å)*		28.63-2.3 (2.36-2.30)*	29.75-2.5 (2.56-2.50)*
Space group		$P3_121$	$P3_121$
Unit cell (Å)	a=b, c (Å)	184, 57.1	184.5, 56.9
	α=β, γ (°)	90, 120	90, 120
Total reflections		98827 (9874)	76521 (7646)
Unique reflections		49419 (4937)	38625 (3842)
Multiplicity		21.13 (3.75)	2.0 (2.0)
Completeness (%)		99.9 (100)	99.79 (100)
Mean I/sigma(I)		15.2 (0.9)	11.80 (3.02)
Wilson B-factor		44.93	47.39
R-merge		0.089 (2.34)	0.113 (1.41)
**Refinement**			
Reflections used in refinement		49411 (4937)	38620 (3842)
R-work		0.22 (0.28)	0.20 (0.26)
R-free		0.25 (0.32)	0.24 (0.29)
Number of non-hydrogen atoms		3444	3384
	macromolecules	3192	3157
	ligands	91	62
	solvent	161	165
RMS (bonds)		0.027	0.034
RMS (angles)		2.37	2.43
Ramachandran favored (%)		93.64	94.61
Ramachandran allowed (%)		5.62	4.9
Ramachandran outliers (%)		0.73	0.49
Average B-factor (Å^2)		57.52	58.58
	macromolecules	57.31	58.55
	ligands	67.12	71.77
	solvent	56.3	54.17

*Values for outermost shell are given in parentheses.

Supplementary Table 2

A. nsp16-RNA cap interactions in the substrate (Cap-0) and product (Cap-1)-bound structures of nsp16/nsp10

		Cap-0 (^{m7}GpppA) [Å]	Cap-1 (^{m7}GpppAmU) [Å]	Deviation [Å]
Nsp16 residue (atom)	Cap residue (atom)			
Leu27 (N)	G_0 (O6)	2.80	3.20	0.40
Tyr30 (OH)	β-phosphate (O21)	2.55	3.19	0.64
	G_0 (C2)	3.41	3.70	0.29
	G_0 (C3)	3.39	4.09	0.70
Lys46 (Nζ)	A_1 (2'-OH)	3.39	5.04	1.65
Tyr132 (OH)	γ-phosphate (O12)	2.56	3.30	0.74
Lys137 (Nζ)	γ-phosphate (O12)	2.88	3.22	0.34
	β-phosphate (O21)	3.01	3.97	0.96
	α-phosphate (O31)	2.63	3.32	0.69
Lys170 (Nζ)	A_1 (2'-OH)	2.79	3.20	0.41
Glu173 (Oε1)	G_0 (C8)	2.97	3.95	0.98
	G_0 (N7)	3.23	4.11	0.88
	G_0 (C7)	3.45	4.03	0.58
Thr172 (Oγ1)	γ-phosphate (O11)	2.65	3.06	0.41
His174 (Cδ2)	G_0 (C5)	3.29	3.95	0.66
	G_0 (O15)	3.25	4.11	0.86
(N)	γ-phosphate (O11)	2.80	3.52	0.72
Asn198 (Nδ2)	A_1 (3'-OH)	3.53	5.14	1.61
Ser201 (Oγ)	α-phosphate (O32)	2.58	3.29	0.71
	β-phosphate (O22)	3.46	3.90	0.44
	α/β-phosphate (O23)	3.70	4.32	0.62
(Cβ)	G_0 (C7)	3.33	4.27	0.94
Ser202 (N)	β-phosphate (O22)	2.79	3.51	0.72
(Oγ)	β-phosphate (O22)	2.76	3.14	0.38
	β/γ -phosphate (O13)	3.00	3.63	0.63
Glu203(Oε1)	A_1 (2'-OH)	3.80	4.96	1.16
	A_1 (3'-OH)	3.70	4.22	0.52

B. Distance between two gate loops in the substrate (Cap-0) and product (Cap-1)-bound structures of nsp16

		Cap-0 (^{m7}GpppA) [Å]	Cap-1 (^{m7}GpppAmU) [Å]	Deviation [Å]
Gate loop 1 residue	Gate loop 2 residue			
Asp26	Asn138	17.20	18.52	1.32
Leu27	Lys137	16.64	18.17	1.53
Gln28	Pro134	23.49	25.44	1.95
Tyr30 (OH)	Lys137 (Nζ)	4.31	5.83	1.52
Gly31	Tyr132	18.17	20.98	2.81

Supplementary Table 3

Sequence of primers used for introducing single point mutations in SARS-CoV-2 nsp16:

Primer name	DNA sequence
S33R-forward	ACTATGGTGATCGTGCGACCCTGCCGAAGGGCATC
S33R-reverse	GGCAGGGTCGCACGATCACCATAGTTTTGCAGGTC
S33N-forward	ACTATGGTGATAACGCGACCCTGCCGAAGGGCAT
S33N-reverse	GGCAGGGTCGCGTTATCACCATAGTTTTGCAGGT
K46A-forward	GAACGTTGCGGCGTACACCCAGCTGTGCCAATAT
K46A-reverse	GCTGGGTGTACGCCGCAACGTTCATCATGATGCC
N198A-forward	GTGGACCGCGTTTGTGACCAACGTTGCGGCGAGCAGCAGCGAGGCGTTCCTGA
N198A-reverse	TCAGGAACGCCTCGCTGCTGCTCGCCGCAACGTTGGTCACAAACGCGGTCCAC

Supplementary References

1. Laskowski R. A., Swindells M. B. (2011). LigPlot+: multiple ligand-protein interaction diagrams for drug discovery. *J. Chem. Inf. Model.*, **51**, 2778-2786.
